# Policy diffusion theory, evidence-informed public health, and public health political science: a scoping review

**DOI:** 10.17269/s41997-023-00752-x

**Published:** 2023-03-21

**Authors:** Katrina Fundytus, Cristina Santamaria-Plaza, Lindsay McLaren

**Affiliations:** 1 Department of Community Health Sciences, University of Calgary, Calgary, AB Canada; 2 School of Epidemiology and Public Health, University of Ottawa, Ottawa, ON Canada

**Keywords:** Public health, Population health, Policy diffusion, Public health policy, Santé publique, santé des populations, diffusion des politiques, politiques de santé publique

## Abstract

**Objectives:**

Our aim was to synthesize published scholarship that applies policy diffusion—a theory of the policy process that considers the interdependence of government-level public health policy choices. We paid particular attention to the role of scientific evidence in the diffusion process, and to identifying challenges and gaps towards strengthening the intersection of public health, public policy, and political science.

**Methods:**

We systematically searched 17 electronic academic databases. We included English-language, peer-reviewed articles published between 2000 and 2021. For each article, we extracted the following information: public health policy domain, geographic setting, diffusion directions and mechanisms, the role of scientific evidence in the diffusion process, and author research discipline.

**Synthesis:**

We identified 39 peer-reviewed, primary research articles. Anti-smoking and tobacco control policies in the United States (*n* = 9/39) were the most common policy domain and geographic context examined; comparatively fewer studies examined policy diffusion in the Canadian context (*n* = 4/39). In terms of how policies diffuse, we found evidence of five diffusion mechanisms (learning, emulation, competition, coercion, and social contagion), which could moreover be conditional on internal government characteristics. The role of scientific evidence in the diffusion process was unclear, as only five articles discussed this. Policy diffusion theory was primarily used by public policy and political science scholars (*n* = 19/39), with comparatively fewer interdisciplinary authorship teams (*n* = 6/39).

**Conclusion:**

Policy diffusion theory provides important insights into the intergovernmental factors that influence public health policy decisions, thus helping to expand our conceptualization of *evidence-informed public health*. Despite this, policy diffusion research in the Canadian public health context is limited.

## Résumé

**Objectifs:**

Nous avons voulu faire une synthèse des travaux d’érudition publiés sur la diffusion des politiques—une théorie du processus d’élaboration des politiques qui prend en considération l’interdépendance des choix de politiques de santé publique au niveau gouvernemental. Nous nous sommes intéressés en particulier au rôle des preuves scientifiques dans le processus de diffusion et à la mise au jour des difficultés et des lacunes associées au renforcement de l’intersection entre la santé publique, les politiques publiques et les sciences politiques.

**Méthode:**

Nous avons systématiquement interrogé 17 bases de données électroniques universitaires. Nous avons inclus les articles en anglais évalués par les pairs publiés entre 2000 et 2021. Pour chaque article, nous avons extrait les informations suivantes : le domaine de politique de santé publique, le lieu géographique, les orientations et les mécanismes de diffusion, le rôle des preuves scientifiques dans le processus de diffusion et la discipline de recherche des auteurs.

**Synthèse:**

Nous avons recensé 39 articles de recherche primaire évalués par les pairs. La lutte contre le tabagisme et les politiques antitabac aux États-Unis (*n* = 9/39) étaient les domaines de politiques et le contexte géographique les plus couramment abordés; comparativement moins d’études portaient sur la diffusion des politiques dans le contexte canadien (*n* = 4/39). En ce qui concerne la façon dont les politiques se diffusent, nous avons relevé cinq mécanismes de diffusion (apprentissage, émulation, compétition, coercition et contagion sociale), qui peuvent de plus dépendre des caractéristiques internes du gouvernement. Le rôle des preuves scientifiques dans le processus de diffusion n’était pas clair, car seulement cinq articles en parlaient. La théorie de la diffusion des politiques était principalement utilisée par les théoriciens des politiques publiques et des sciences politiques (*n* = 19/39), avec comparativement moins d’équipes d’auteurs interdisciplinaires (*n* = 6/39).

**Conclusion:**

La théorie de la diffusion des politiques apporte des éclairages utiles sur les facteurs intergouvernementaux qui influencent les décisions en matière de politiques de santé publique, ce qui contribue à élargir notre conceptualisation de la *santé publique éclairée par les données probantes*. Malgré cela, la recherche sur la diffusion des politiques dans le contexte de la santé publique canadienne est limitée.

## Introduction

There is long-standing, yet under-mobilized, recognition that governments can influence the distribution of the social determinants of health and health inequities (i.e., unfair and avoidable differences in health outcomes) by enacting public policies in domains such as housing, employment, and environment (Hancock, 1985; Raphael, 2020; World Health Organization, 2010). Public policies broadly refer to the decisions (both action and inaction) of a government, and can include statutes, regulations, procedures, programs, and executive decisions (Weible, 2014).

Public policy decision-making is complex, and one approach to better understand the intricacies of policymaking is to consider theories of the policy process (Cairney & Oliver, 2017; Fafard, 2015; Fafard & Cassola, 2020). The present study focuses on *policy diffusion*, where policy decisions in one jurisdiction influence policymaking in other jurisdictions (Berry & Berry, 2014). Policy diffusion is anchored in the recognition that policy adoption is inherently interdependent, and rarely occurs as a result of internal factors alone (Berry & Berry, 2014; Petridou, 2014).

Policy diffusion is a distinct class of studies within a broader literature on innovation and diffusion (Shipan & Volden, 2012). It draws heavily from Everett Rogers’ diffusion of innovations theory (Rogers, 1962, 2003), which examines the spread of non-policy innovations (i.e., individual- or organization-level interventions) via communication channels over a range of areas (e.g., teaching practices in school systems, medical/health ideas in hospitals). Scholarship in policy diffusion has evolved to incorporate new approaches and techniques that build upon Roger’s original framework (Berry & Berry, 2014, 2018; Karch, 2022). The present work is situated within this contemporary scholarship as described next.

Policy diffusion theory has been used to study whether, how, and why policies spread across government jurisdictions. This can occur in four directions: *horizontal*, diffusion across the same government level (e.g., provincial-to-provincial); *bottom-up vertical*, occurs from lower- to higher-level governments (e.g., local-to-provincial); *top-down vertical*, policy spreads from a higher- to lower-level government (e.g., provincial-to-local); and, *replication*, where a single government applies existing policy ideas to a new analogous policy domain (e.g., policy ideas spread across different domains within the same government) (Shipan & Volden, 2006; Train & Snow, 2019). In addition, five key mechanisms of diffusion have been identified (Berry & Berry, 2014; Maggetti & Gilardi, 2016; Pacheco, 2012; Shipan & Volden, 2008). Briefly, *learning* is when policymaking in one jurisdiction is influenced by the observed consequences of policies in other jurisdictions; the more successful a policy, the more likely its adoption elsewhere. Unlike learning, *emulation* is not contingent on whether a policy “works”; policy decisions are instead influenced by the normative environment or social acceptability. *Coercion* occurs when one government pressures others to take policy action via threat or incentive. *Competition* occurs when policy decisions are made to gain economic advantage (or avoid disadvantage) over other jurisdictions. Finally, *social contagion* refers to policy learning at the citizen level (as opposed to the government level), and the corresponding policy responsiveness of government officials.

Although there is a large literature on policy diffusion theory in political science and policy studies (Berry & Berry, 2014; Graham et al., 2013), its application to public health policy is not well studied (Breton & de Leeuw, 2011; Moloughney, 2012). This presents an important knowledge gap, which is perhaps indicative of a broader interdisciplinary research challenge identified by scholars working at the intersection of political science, public policy, and public health (Fafard & Cassola, 2020). Specifically, within the public health literature, only a limited number of theories of the policy process have been cited (Breton & de Leeuw, 2011; Cairney, 2016; Cairney et al., 2016, 2022; Moloughney, 2012), and the application of these theories tends to be superficial or descriptive (Breton & de Leeuw, 2011; Clarke et al., 2016; Moloughney, 2012).

Public health scholarship often endorses (implicitly or explicitly) a linear evidence-to-policy model of policy decision-making, where scientific evidence flows directly from knowledge producer (i.e., researchers) to users (i.e., policymakers) (Cairney, 2016; Fafard & Hoffman, 2020; Fafard et al., 2022). Evidence-informed public health (EIPH) is an example of this model (National Collaborating Centre for Methods and Tools, 2018). In contrast to the evidence-to-policy model, important scholarship has identified that the production and dissemination of scientific evidence alone does not have substantive impact on public policymaking (Cairney, 2016; Cairney & Oliver, 2017; Fafard & Cassola, 2020). Although scientific evidence can help to reduce uncertainty (i.e., lacking information on a policy problem), it does little to reduce ambiguity (i.e., lacking agreement on how to define/frame a policy problem) (Cairney, 2016; Cairney et al., 2022). To resolve ambiguity, policymakers draw upon different forms of “evidence” (e.g., value judgements, public opinion, “expert” consultation, emotions) to legitimize how policy problems are framed or prioritized (Cairney, 2016; Cairney & Oliver, 2017; Cairney et al., 2016; Oliver, 2022). Moreover, although often perceived as apolitical, the production, interpretation, and use of scientific evidence are value-based, contested, and influenced by structures of politics and power (Cassola et al., 2022; Parkhurst, 2017).

The learning mechanism of policy diffusion explicitly focuses on identifying indicators of policy success and effectiveness, which can include (but is not limited to) scientific evidence (Cairney, 2016; Olive & Boyd, 2021; Shipan & Volden, 2008). However, measures of success or effectiveness are rarely clear, can vary between governments, and are often based on limited scientific evidence (Cairney, 2016; Shipan & Volden, 2012). Overall, policy diffusion is not a technocratic process, but instead involves varied measures of policy success, value judgements, assessments of policy compatibility, and political considerations (Cairney, 2016; Olive & Boyd, 2021). We therefore seek to identify the role of scientific evidence in the policy diffusion process, and whether this differs across the diffusion mechanisms.

Overall, our aim is to identify and synthesize published, peer-reviewed scholarship that applies policy diffusion theory to public health policy (defined as a subset of public policies that aim to improve the health of populations), with particular attention to the role of scientific evidence in the diffusion process. We also aimed to identify challenges and gaps for research at the intersection of political science, public policy, and public health. To do so, we posed four research questions of the peer-reviewed literature:

1. In what geographic settings and public health policy domains has policy diffusion theory been used or applied?
2. How common are the five mechanisms identified in policy diffusion theory in the diffusion of public health policy?
3. What role does scientific evidence play in policy diffusion, and how does this relate to the five mechanisms, if at all?
4. To what extent is there cross-disciplinary engagement with diffusion theory in public health policy, particularly between public health, public policy, and political science?

## Methods

We undertook a scoping review, following methods described by Arksey and O’Malley (2005) and the Preferred Reporting Items for Systematic Reviews and Meta-Analysis extension for Scoping Reviews (Tricco et al., 2018). The aim of a scoping review is to identify what is known about a particular concept (i.e., the application of diffusion theory to public health policies) and types of available evidence (Arksey & O’Malley, 2005).

### Data sources and search strategy

**Fig. 1:**
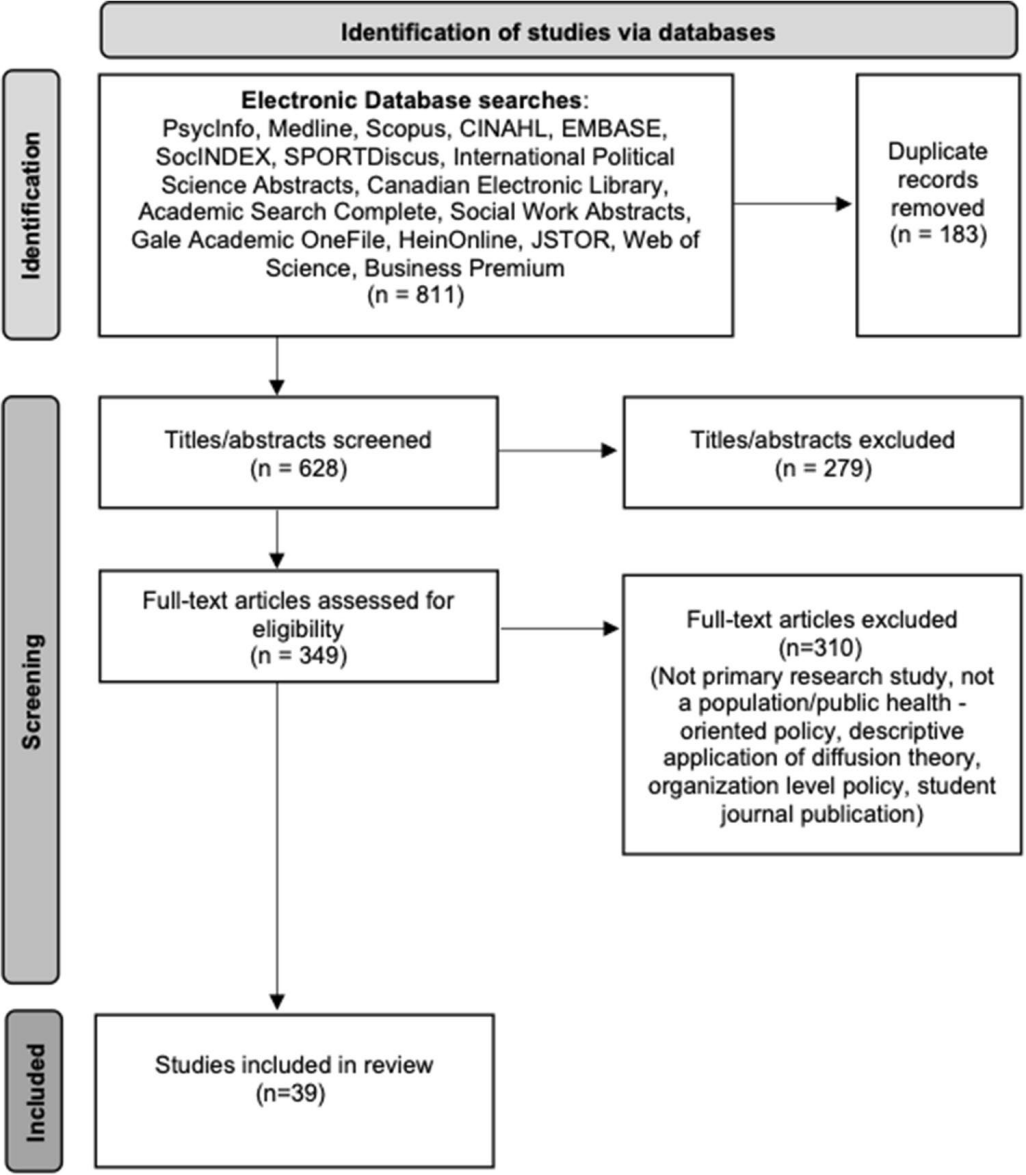
PRISMA flow diagram of included articles

We systematically searched 17 electronic academic databases for peer-reviewed, English-language articles (see Fig. 1 (PRISMA) for the full list of academic databases). We used the search terms “policy diffusion” and (“population health” or “health promotion” or “public health”) in the article’s subject heading, title, abstract, keyword, or full text. As noted above, scholarship in the 1990s highlighted significant flaws in traditional methodologies of diffusion and innovation, and with the introduction of new empirical techniques, newer approaches have emerged and strengthened (Berry & Berry, 2014, 2018; Karch, 2022). We considered articles published between January 1, 2000, and June 20, 2021, to focus primarily on this contemporary era of diffusion theory.

Two authors (KF and CSP) independently screened citation abstracts and titles using Covidence reference management software (Covidence, 2021). Full-text versions of all potentially relevant citations were independently reviewed by the same two authors, through which a final list of articles was compiled for extraction and analysis.

### Inclusion and exclusion criteria

We only considered primary research; reviews and commentaries were excluded. We also excluded books, book chapters, conference papers, abstracts, and student journals. Articles had to go beyond description of policy diffusion to integrate key concepts into a framework and/or to guide data collection and analysis (adapted from Breton & de Leeuw, 2011). In other words, articles that described diffusion but lacked an explicit application of diffusion theory, were excluded.

As per our definition of public policy (above), we only considered articles that focused on policy diffusion across a discrete political system (i.e., local, subnational, national, international) as opposed to a smaller, organizational level of governance such as schools, workplaces, or hospitals. “Public health policy” is different from “health (care) policy” and we excluded articles that focused on a healthcare-oriented policy (Dalla Lana School of Public Health, n.d.). Articles had to describe the implications of the public policy for population health outcomes. This permitted us to embrace a broad definition of “public health policy”, which included (for example) infectious disease prevention (e.g., vaccination) and tobacco control, as well as broader social policies, such as gun control or animal regulation.

### Analysis

Guided by our research questions, we collated key information from each article using a coding template developed iteratively by KF and LM throughout the full-text article extraction phase. The following information was recorded for each article: publication year, general study design (i.e., quantitative, qualitative, mixed methods), journal title, key objectives and/or hypotheses, results in relation to policy diffusion theory, policy setting (i.e., primary geographic location), level of government (i.e., local, subnational, national, or international), policy domain (i.e., policy area), and diffusion direction (i.e., horizontal, bottom-up, top-down, replication). We also recorded information on the mechanisms of diffusion (i.e., learning, emulation, coercion, competition, and social contagion), including how these were defined and operationalized.

Finally, against the backdrop of evidence-informed public health, and a lack of integrated research partnerships between public health, political science, and public policy scholars, we recorded information on (1) the role of scientific evidence in policy diffusion, and in relation to the diffusion mechanisms specifically (if discussed), and (2) each author’s scholarly discipline (based on their formal academic training and academic department appointment) to gauge the extent of interdisciplinary research teams.

## Results

From an initial set of 628 articles, of which 349 were deemed potentially relevant based on title/abstract, we ultimately analyzed 39 peer-reviewed research articles that applied policy diffusion theory to a public health policy (see Fig. 1 (PRISMA), and Table 1 for descriptive study characteristics).

**Table 1:** Summary table of descriptive study characteristics

Study	Author research discipline(s)	Study design	Geographic setting	Government-level; diffusion direction	Policy domain	Diffusion mechanism(s)	Did policy diffusion occur?	Referenced scientific evidence
Agostinis, 2019	Political science	Qualitative	Various	International; horizontal	Various (cancer control and public health education)	Learning*	Yes	No
Anderson et al., 2016	Public administration, public health policy, engineering, community health sciences	Quantitative	USA	Subnational; horizontal	Various (alcohol, driver safety, impaired driving)	N/A	Yes	No
Bessho, S. & Ibuka, Y., 2018	Economics	Quantitative	Japan	Local; horizontal	Vaccination	N/A	Yes	No
Boehmke, F., 2009	Political science	Quantitative	USA	Subnational; horizontal	Obesity	N/A	Mixed; methods paper outlining different approaches to model policy diffusion	No
Boyle et al., 2015	Sociology	Quantitative	Various	International; horizontal	Abortion liberalization	N/A	Yes, typical and atypical	No
Chorev, 2012	Sociology	Qualitative	Various	International; horizontal	HIV/AIDS	Learning*	Yes	No
Clark, 2013	Public policy, public administration	Quantitative	Various	International; horizontal	HIV/AIDS	Learning	Mixed; role of diffusion is not clear	No
Clark, 2009	Public policy, public administration	Quantitative	Various	International; horizontal	HIV/AIDS	N/A	Yes, atypical	No
Clouser-McCann et al., 2015	Political science, public health, public policy	Quantitative	USA	National, subnational, local; horizontal, vertical (bottom-up), vertical (top-down)	Anti-smoking/tobacco	N/A	Yes	No
Curiel et al., 2020	Political science, public health dentistry, dental ecology	Quantitative	USA	Local; horizontal	Community water fluoridation	Emulation*	Yes	No
Fix & Mitchell, 2017	Political science	Quantitative	USA	Subnational; horizontal	Local breed legislation	Learning*	Yes	No
Givens & Mistur, 2021	Political science, public administration, public policy	Quantitative	Various	International; horizontal	COVID-19	Emulation*	Yes	Yes
Godwin, M. & Schroedel, JR, 2000	Political science, public administration	Mixed methods	USA	Local; horizontal	Gun control	N/A	Yes	No
Johns, 2015	Sociology, political science	Mixed methods	USA	Local; replication	Marijuana	Learning*	Yes	No
Johnson, B. & White, S., 2010	Political science, urban planning, land resources	Qualitative	USA	Local; horizontal	Sustainable transportation infrastructure	Learning*	Yes	No
Kadowaki et al., 2015	Sociology	Quantitative	USA	Subnational, local; horizontal, vertical (bottom-up)	e-cigarette restrictions	N/A	Inconclusive	Yes
Kavanagh et al., 2021	Political science, international health	Mixed methods	Various	International; horizontal	HIV/AIDS	Learning	No	Yes
Macinko & Silver, 2015	Health policy, public health policy, public administration	Quantitative	USA	Subnational; horizontal, replication	Impaired driving	Learning	Yes	Yes
Mallinson, 2016	Political science, public policy	Quantitative	USA	Subnational; horizontal	Anti-bullying measures	Learning*	Yes, atypical	No
Michael, 2016	Political science	Mixed methods	Various	International; horizontal	Pharmacuticals	Coercion* Competition Emulation Learning	Yes	No
Mitchell & Stewart, 2014	Political science, public policy, public administration	Quantitative	USA	Local; horizontal	Anti-smoking/tobacco	Competition* Emulation Learning*	Yes	No
Moreland-Russell, S. et al., 2013	Public health, public policy, community health, social ecology	Quantitative	USA	Local; horizontal	Complete streets policies	N/A	Yes	No
Nykiforuk, C. et al., 2018	Public health, health promotion	Quantitative	Canada	Local; horizontal	Fast food bylaw	Learning	Yes	No
Nykiforuk, C. et al., 2018	Public health, health sciences, geography	Quantitative	Canada	Subnational, local; horizontal, vertical (top-down)	Anti-smoking/tobacco	N/A	Yes	No
Olstad et al., 2015	Public health, nutrition	Qualitative	Canada	Subnational; horizontal	Daily physical activity	N/A	Yes	Yes
Pacheco, 2017	Political science, public policy	Quantitative	USA	Subnational; horizontal	Anti-smoking/tobacco	Competition*	Yes, typical and atypical	No
Pacheco, 2012	Political science, public policy	Quantitative	USA	Subnational; horizontal	Anti-smoking/tobacco	Competition Learning Social contagion*	Yes	No
Pacheco & Boushey, 2014	Political science, public policy	Quantitative	USA	National, subnational; horizontal, vertical (top-down)	Various (anti-smoking/tobacco, vaccination)	N/A	Yes	No
Sebhatu et al., 2020	Sociology, business, political science	Quantitative	Various	International; horizontal	COVID-19	Emulation*	Yes	No
Septiono, W. et al., 2019	Public health	Quantitative	Indonesia	Subnational, local; horizontal, vertical (top-down)	Anti-smoking/tobacco	N/A	Yes	No
Shen, 2014	Public health, health policy	Quantitative	Various	International; horizontal	Mental health	Coercion Emulation*	Yes	No
Shipan & Volden, 2008	Political science, public policy	Quantitative	USA	Subnational, local; horizontal, vertical (top-down)	Anti-smoking/tobacco	Coercion* Competition* Emulation* Learning*	Yes	No
Shipan & Volden, 2006	Political science, public policy	Quantitative	USA	National, subnational, local; horizontal, vertical (bottom-up), vertical (top-down)	Anti-smoking/tobacco	N/A	Yes	No
Shipan & Volden, 2014	Political science, public policy	Quantitative	USA	Subnational; horizontal	Anti-smoking/tobacco	Learning*	Yes	No
Sieger, M. & Rebbe, R., 2020	Social work	Quantitative	USA	Subnational; horizontal	Child abuse prevention and treatment act	N/A	Yes	No
Train & Snow, 2019	Political science	Qualitative	Canada	National, subnational; replication, horizontal, vertical (top-down)	Marijuana	Coercion* Competition Emulation Learning*	Yes	No
Trein, 2017	Political science	Quantitative	Switzerland	Subnational; horizontal	Anti-smoking/tobacco	Social contagion*	Yes	No
Valente, T. et al., 2015	Public health, communication, sociology, communication and information sciences, international relations	Quantitative	Various	International; horizontal	Anti-smoking/tobacco	N/A	Yes	No
Winder & LaPlant, 2000	Political science	Quantitative	USA	Subnational; horizontal	Anti-smoking/tobacco	N/A	Mixed; report diffusion but no consistent regional pattern	No

*Mechanism significant

**Atypical = slowed/decreased government action

### Public health policy geographic settings, government level, and diffusion direction

Most articles focused on policies in the United States (*n* = 21/39) or, to a much lesser extent, Canada (*n* = 4/39). Other primary settings included Japan, Indonesia, and Switzerland. In terms of government level, the most common were subnational (e.g., province, canton, state) (*n* = 20/39) and local level (e.g., county, municipality) (*n* = 14/39); international policy diffusion (i.e., country-to-country) (*n* = 11/39) was also common. For diffusion direction,^1^ nearly all articles examined horizontal diffusion (*n* = 38/39), with notably fewer examining top-down (*n* = 7/39), bottom-up (*n* = 3/39), or replication (*n* = 3/39) (Table 1).

### Public health policy domains and evidence of diffusion

Policy diffusion was applied to several public health domains, most commonly anti-smoking- and tobacco-related policies (*n* = 13/39) (e.g., Shipan & Volden, 2006) and HIV/AIDS-related policies (*n* = 4/39) (Chorev, 2012; Clark, 2013; Clarke et al., 2016; Kavanagh et al., 2021). Other policy domains included COVID-19 (*n* = 2/39) (Givens & Mistur, 2021; Sebhatu et al., 2020), marijuana (*n* = 2/39) (Johns, 2015; Train & Snow, 2019), vaccinations (*n* = 2/39) (Pacheco & Boushey, 2014), and impaired driving (*n* = 2/39) (Anderson et al., 2016; Macinko & Silver, 2015) (see Table 1 for full list of policy domains).

Although assessment of whether diffusion occurred or not is complicated by different research questions and methods, we ultimately identified that most (*n* = 34/39) articles showed evidence of policy diffusion. For example, in the USA, Shipan and Volden (2006) found that the likelihood of state-level governments adopting an anti-smoking policy increased as neighbouring states passed such policies. In the Canadian context, all four articles^2^ demonstrated the role of policy diffusion in the adoption and spread of school-based daily physical activity policies (provincial) (Olstad et al., 2015), fast food drive-through and smoking restriction bylaws (local) (Nykiforuk et al., 2008, 2018), and recreational marijuana regulation (provincial) (Train & Snow, 2019).

Four articles found an atypical pattern of diffusion, where neighbouring policy adoption slowed or decreased the likelihood of local policy adoption, in the policy domains of tobacco control (Pacheco, 2017), anti-bullying (Mallinson, 2016), abortion liberalization (Boyle et al., 2015), and HIV/AIDs (Clark, 2009). For example, Clark (2009) identified that as the proportion of AIDS program adoption in geographically neighbouring countries increased, the time leading to local adoption also increased. In contrast, several articles (*n* = 5/39) found mixed, inconclusive, or nonsignificant evidence of diffusion. For example, Kavanagh et al. (2021) identified formal government structures and racial stratification as better predictors of HIV treatment policy adoption compared to the policy choices of neighbouring governments.

### The mechanisms of public health policy diffusion: learning, emulation, competition, coercion, and social contagion

Just over half of the articles (*n* = 22/39) referenced at least one mechanism of diffusion. The most common was learning (*n* = 16/39), then emulation (*n* = 8/39), competition (*n* = 6/39), coercion (*n* = 4/39), and social contagion (2/39).^3^ There was heterogeneity in terms of how the diffusion mechanisms were measured or conceptualized, with different indicators used for the same mechanism across the included articles.^4^ For example, in the case of policy learning, measurements ranged from broad-level indicators, such as the number of bordering governments that adopted a policy the previous year (Mitchell & Stewart, 2014), to more specific indicators, such as demonstrated success of a policy adopted by a government elsewhere (Shipan & Volden, 2014) or explicit reference to another government as a source of information and legitimacy (Chorev, 2012).

Notwithstanding these different ways of measuring each mechanism, there were examples of each occurring, which varied by geographic context and policy domain. Policy learning was evident in ten articles, including the adoption of cancer control policies and public health training in South America (Agostinis, 2019), youth tobacco restriction policy adoption in the USA (Shipan & Volden, 2014), intellectual property rights of AIDS drugs (Chorev, 2012), and dog breed specific legislation in the USA (Fix & Mitchell, 2017). Two articles identified the role of the learning mechanism via replication diffusion in marijuana regulation (Johns, 2015; Train & Snow, 2019); for example, a greater number of American cities in the state of Colorado permitted the sale of recreational marijuana if they had previously implemented a medical marijuana-use policy (Johns, 2015).

Emulation was significant in the adoption of COVID-19 policies (Givens & Mistur, 2021; Sebhatu et al., 2020) and mental health policy (Shen, 2014) internationally, and local-level anti-smoking (Shipan & Volden, 2008) and community water fluoridation policies^5^ (Curiel et al., 2020) in the USA. For example, one study identified that “nationalist”^6^ countries were more likely to implement a policy change the day after a country with a similar nationalist regime changed its respective COVID-19 policies (Givens & Mistur, 2021). At the local level of government in the USA, Shipan and Volden (2008) found American cities more likely to adopt an anti-smoking law when the nearest, largest neighbouring city had previously adopted such a law.

Competition was evident in anti-smoking and tobacco control policies in the USA at the local (e.g., clean indoor air laws, youth access policies) (Mitchell & Stewart, 2014; Shipan & Volden, 2008) and state levels (e.g., tobacco sale and consumption) (Pacheco, 2017). Pacheco (2017) identified two ways that competition can influence tobacco and anti-smoking policy at the state-level in the USA: competitive races (i.e., policy changes in one jurisdiction encourage others to adopt similar policies to gain economic or other benefits) and free-rider dynamics (i.e., positive spillover effects of a policy in one jurisdiction incentivize others not to adopt).

Coercive pressures contributed to policy adoption in the domains of marijuana regulation (Train & Snow, 2019), intellectual property right laws (Michael, 2016), and anti-smoking and tobacco (Shipan & Volden, 2008). One study examined the global diffusion of intellectual property right agreement laws for pharmaceutical clinical trial data; it identified that powerful countries can dictate the terms of these laws to other countries by threatening to withhold benefits during trade negotiations (Michael, 2016). In Canada, coercive pressures from the federal government influenced the diffusion of marijuana legalization at the provincial level in Ontario and New Brunswick by placing heavy constraints on provincial autonomy to regulate the production, distribution, sale, and consumption of cannabis (Train & Snow, 2019).

Finally, two articles, both in the anti-smoking and tobacco domain, reported evidence of social contagion (Pacheco, 2012; Trein, 2017). Pacheco (2012) identified that public opinion of restaurant smoking bans is influenced by the policy decisions in neighbouring states; if state-wide opinion becomes supportive of these bans, officials then respond by enacting similar policies locally.

### Internal government characteristics and policy diffusion mechanisms

Diffusion mechanisms sometimes overlapped in the same policy domain or geographic setting (Mitchell & Stewart, 2014; Shipan & Volden, 2008; Train & Snow, 2019). Moreover, they were sometimes contingent on internal government characteristics, such as government regime (Givens & Mistur, 2021; Sebhatu et al., 2020), policy expertise (Shipan & Volden, 2014), legislative professionalism (Pacheco & Boushey, 2014; Shipan & Volden, 2014), and policy problem severity (Fix & Mitchell, 2017). For example, in the USA, states with a higher number of dog fight cases or fatalities from dog bites (i.e., high problem severity) were more likely to adopt breed-specific legislation, compared to states with lower numbers (Fix & Mitchell, 2017). Conversely, Givens and Mistur (2021) did not find a consistent significant relationship between policy problem severity (in the form of COVID-19 cases per capita) and the adoption of COVID-19 policies by “nationalist” countries.

### Scientific evidence in public health policy diffusion and the policy diffusion mechanisms

The role of scientific evidence in the policy diffusion process was not frequently examined. Five studies discussed scientific evidence in some capacity (*n* = 5/39) (Givens & Mistur, 2021; Kadowaki et al., 2015; Kavanagh et al., 2021; Macinko & Silver, 2015; Olstad et al., 2015). Only three articles (*n* = 3/5) referenced at least one policy diffusion mechanism and scientific evidence; however, none of these articles empirically examined the role of scientific evidence in relation to the diffusion mechanisms.

In one article, Givens and Mistur (2021) interpreted the observed pattern of COVID-19 policy adoption by “nationalist” countries (see above) as suggesting that these governments “emulate” the policies of other countries with similar nationalist regimes, instead of following scientific evidence. In another article, Macinko and Silver (2015) examined the role of policy learning (via replication)^7^ and other determinants in evidence-based impaired driving law adoption in the USA; although the authors assert more generally that patterns of state-level health policy adoption ought to be understood as more than a direct response to emerging evidence, this was not explicitly examined in their analysis. Finally, one article empirically considered the role of scientific evidence and policy learning in global HIV treatment policy decision-making, but neither were found to be strong or consistent indicators of policy adoption (Kavanagh et al., 2021).

Two articles discussed scientific evidence more broadly but did not examine any diffusion mechanisms in their analysis (Kadowaki et al., 2015; Olstad et al., 2015). Kadowaki et al. (2015) identified a spatially uneven pattern of adoption of state- and local-level e-cigarette clean air policies in the USA, and partially attributed this to policy needs outpacing available scientific evidence, and a general lack of consistent scientific evidence creating confusion among policymakers. In the Canadian context, Olstad et al. (2015) identified that provincial governments (Alberta, British Columbia, Manitoba, and Saskatchewan) cited an international body of evidence as a rationale for adopting daily physical activity policies for children. However, it was not clear whether or the extent to which this evidence informed the specific provisions of each province’s policy; provincial policies varied across the country, and in some cases, did not coincide with the established national guidelines.

### Cross-disciplinary engagement with diffusion theory: public health, public policy, and political science

The majority of authorship teams on studies included in our review consisted of scholars from the political science, public policy, and public administration research domains only (*n* = 19/39). There were fewer cross-disciplinary research teams consisting of both public health and political science or public policy scholars (*n* = 6/39), and even fewer consisting of public health scholars only (*n* = 3/39). Other research disciplines included sociology (*n* = 3/39), social work (*n* = 1/39), and economics (*n* = 1/39) (see Table 1).

## Discussion

Policy diffusion theory highlights the importance of considering the interdependence of public health policy decisions. We found that application of the theory is particularly developed in the domain of anti-smoking and tobacco policy in the USA. Comparatively, there were relatively fewer articles in the Canadian context, which examined a range of policy domains and levels of government (Nykiforuk et al., 2008, 2018; Olstad et al., 2015; Train & Snow, 2019).

Despite recognition of the importance and relevance of policy diffusion research by Canadian researchers (Place Research Lab, n.d.; Politis et al., 2014), we found few examples of public health policy diffusion scholarship in the Canadian context, consistent with findings elsewhere (Olive & Boyd, 2021). Our findings build on existing public health policy diffusion scholarship in Canada (Campbell et al., 2020; Nykiforuk et al., 2008, 2018; Olstad et al., 2015; Place Research Lab, n.d.), which primarily adapts Roger’s diffusion of innovations theory to explain adoption patterns (Rogers, 2003). Although Roger’s theory is widespread in health sciences and healthcare innovation research, our review captures contemporary policy diffusion scholarship to include (for example) Berry and Berry (1990), Maggetti and Gilardi (2016), Shipan and Volden (2008), and Volden (2006).

We found evidence of five mechanisms of diffusion (i.e., learning, emulation, competition, coercion, and social contagion), which vary depending on policy domain, geographic context, and internal government characteristics. Our findings show that local public health problem severity (e.g., motor vehicle fatalities, COVID-19 cases) is not a reliable predictor of policy action (Givens & Mistur, 2021; Kavanagh et al., 2021; Sebhatu et al., 2020; Winder & LaPlant, 2000). From the perspective of public health practice, this finding confirms tacit understanding that public health surveillance, while important and necessary, is not sufficient to prompt public policy action (Chambers et al., 2006). Governments may vary in their capacity to obtain, analyze, and use this information (Clouser-McCann et al., 2015; Shipan & Volden, 2014), or governments may be aware of public health threats, but privilege other factors in decision-making, such as non-health measures of policy success (Shipan & Volden, 2008), or pressures from other government jurisdictions via one or more diffusion mechanisms.

Evidence-to-policy models in public health often assert that improved knowledge translation efforts (i.e., researchers more effectively providing policy decisionmakers with scientific evidence) will increase the likelihood that scientific evidence will inform policy decisions (Fafard, 2008). However, this is not well supported by our findings. Scientific evidence was either absent or did not play a significant role in policy diffusion more generally, or across the five diffusion mechanisms. Even when policymakers are aware of and able to articulate pertinent scientific evidence (Kavanagh et al., 2021), they may privilege other factors in policy decisions. In the case of policy learning, for example, instead of engaging directly with scientific evidence, governments may look for other indicators of policy success, such as widespread policy adoption without subsequent abandonment across other jurisdictions (Shipan & Volden, 2008). Thus, there is a need for public health research to consider what constitutes appropriate and relevant evidence in the policy diffusion process, and in relation to each of the diffusion mechanisms, as opposed to what “should” inform policymaking based on established hierarchies that favour certain types of scientific evidence (e.g., systematic reviews, randomized controlled trials) and their accompanying epistemological perspectives (Oliver, 2022; Parkhurst, 2016).

Finally, despite the complementary nature of political science, public policy, and public health disciplines, we found little evidence of interdisciplinary research partnerships (*n* = 6/39), with most article authors having formal academic training in political science or public policy studies. To address this challenge, scholars have emphasized the need for a more collaborative approach to public health policy analysis, termed “public health political science” (Fafard & Cassola, 2020; Greer et al., 2017). Public health political science seeks to incorporate insights from public health, public policy, and political science to provide a more robust approach to address politics, political systems, and the public health policy process (Fafard & Cassola, 2020; Greer et al., 2017). Based on the relatively low number of cross-disciplinary research teams in our sample, we see this as an important area of growth in public health policy scholarship.

This scoping review has several limitations. First, we only considered articles that used the term “policy diffusion” and did not include related terms such as policy transfer or convergence in our database search; these terms—though related and complementary—are distinct research areas, and we therefore maintained our conceptual focus on policy diffusion (Gilardi & Wasserfallen, 2019; Graham et al., 2013; Petridou, 2014; Shipan & Volden, 2012). Nonetheless, our omission of these related subfields may underrepresent the number of articles that examine government-level public health policy interdependence, as well as the extent of interdisciplinary engagement with this literature.

Second, as this is a scoping review, we did not assess the quality or rigour of the included studies. In terms of strengths, we highlight our systematic approach to identify relevant peer-reviewed articles, and in particular, our comprehensive search of 17 electronic databases, and the use of two authors to screen abstracts and full-text articles. Moreover, this is the first review to examine the application of policy diffusion theory to government-level public health policy specifically; historically, policy diffusion theory has not been included in reviews on the application of policy process theories in public health research (Breton & de Leeuw, 2011; Moloughney, 2012). Directions for future research could include (but are not limited to) examining the specific role(s) of scientific evidence and other “types” of evidence in relation to the five mechanisms of policy diffusion, and across different public health policy domains and geographic contexts.

## Conclusion

Policy diffusion theory has relevance to public health policy scholarship for two key reasons. First, and more generally, the use of political science and policy process theory in public health scholarship is rare, and focusing on policy diffusion provides one example of the richness and nuance that can come from applying a theory of the policy process to public health policy scholarship. Second, policy diffusion specifically is informative for public health policy because it can lead to both positive and negative consequences for public health outcomes, which may be missed if the primary focus is on scientific evidence (as per evidence-informed public health, for example). It illuminates the policy decisions of other governments as a key source of information, which may be in addition to, or instead of, scientific evidence and internal factors. The effect of policy diffusion can be positive if, for example, governments learn about effective public health policies from other governments, which can save both time and resources (Place Research Lab, n.d.). Conversely, through policy diffusion processes, the wrong lessons can be learned from others’ experiences, or governments may feel pressured to conform to the policy decisions of other “like-minded” governments even if they are “ineffective”, or they may seek to establish a competitive advantage over others (Shipan & Volden, 2012). Thus, the study of how and why, via the key mechanisms, policies diffuse has relevance to understanding what factors, aside from scientific evidence, contribute to public health policy decision-making and ultimately to public health outcomes such as population health status and health inequities.

## Data Availability

Data are available from the authors upon reasonable request.

## Footnotes

1 Several papers examined more than one level of government and/or diffusion direction.

2 Three articles (3/4) in the Canadian context applied Roger’s Diffusion of Innovations framework (Rogers, 2003).

3 Six articles examined more than one mechanism in the same policy area (Michael, 2016; Mitchell  & Stewart, 2014; Pacheco, 2012; Shen, 2014; Shipan & Volden, 2008; Train & Snow, 2019).

4 This is an identified limitation of the policy diffusion literature more broadly (Maggetti & Gilardi, 2016).

5 Although in this case, the emulation mechanism bears strong resemblance to the learning mechanism as it is described in other studies.

6 Here, Givens and Mistur (2021) define nationalism as “…a malleable and narrow ideology that values membership in a nation more highly than belonging to other groups… seeks distinction from other nations, strives to preserve the nation, and gives preference to political representation by the nation for the nation.” (pp.215); nationalism was measured using Varieties of Democracy (V-Dem) nationalism scores.

7 Policy learning via replication was found to be a non-significant predictor of state-level impaired driving laws adoption in the USA.
